# Early Experiences and Reminiscences of Forty Years of Practice

**Published:** 1898-07

**Authors:** G. G. Roy

**Affiliations:** Atlanta, Ga., Professor Materia Medica and Therapeutics, Southern Medical College


### EARLY EXPERIENCES AND REMINISCENCES OF FORTY YEARS OF PRACTICE.

By G. G. ROY, M.D., ATLANTA, GA.,

Professor Materia Medica and Therapeutics, Southern Medical College.

About midnight during the first year of my active professional life a negro messenger rapped at my office door, arousing me from profound slumber (not from exhausted mental and physical forces from *overwork*, but more likely from the buoyancy of young manhood and the happiness of anticipated fame and fortune, not unusual dreams of young physicians *at the start*), and announced that Aunt Nancy (the negro midwife) had sent for me to see Lizzie (negro woman), and said I must "come in a hurry, because Lizzie was in the most curious fix she had ever seed any 'oman sence she bin ketchin' babies."

I questioned the boy, hoping to get an idea of the woman's condition, in order to review in my memory all the books I had read touching such a case, and have my plans well laid for treatment by the time I reached the patient. And just here I would throw out a hint to my young brethren: It is a good plan to get all the information about your patient's symptoms before you get to the house, and review them in your mind; jog your memory and bring back everything you ever read or heard of bearing on such symptoms; it will make you more self-possessed and calmer when you enter the sick-chamber, even if you find yourself confronted with a serious and puzzling case. Nothing hurts a young doctor more than to be flurried and hesitating about what to do, in the minds of the ignorant attendants, when he approaches the bedside of a patient.

The boy said: "De Lord, doctor! I don't know what's de matter wid Lizzie. I just heard Aunt Nancy a-telling de 'omens standing 'round dat Lizzie was trying to have a baby, and de baby and de whole substunce in her done come into de world, and de baby and de whole substunce is lyin' on de bed 'twixt Lizzie knees."

I said: "What! the child and the womb all out on the bed down between her knees?"

"Dat's just what Aunt Nancy tell de 'omens."

Now you better believe I had food for reflection during that hour I was reaching my patient.

I had never seen, or read of such a thing in the books. The nearest approach to it that my mind could "conjure up" was the cut in Professor Chas. D. Meegs's Obstetrics of a case of procidentia uteri. There the vagina is completely inverted and the womb is exposed. "But," thought I, "that is an unimpregnated womb. I wonder why Dr. Meegs didn't say something on what to do with a womb with the child in it?"

I rode on, hoping and praying that Aunt Nancy was mistaken and things were not so bad. I reached the house and entered the room of my patient with a sinking heart but a bold face.

The negroes had put out the "noration," and about all the women on the large plantation had gathered in and around Lizzie's house.

Aunt Nancy was seated by the bedside, swaying herself backward and forward in a chair (like negroes do at a camp-meeting), with a face as long as my arm. She arose quickly, took me outside the house into a chimney-corner, and began to tell me her experience with the case, winding up by saying: "I neber seed such a case, doctor, in all my born days. Please come into de house and try to do sumting for dat gal."

I went in, removed the covering, and saw just what Aunt Nancy and the boy had told me was true. There lay the womb, holding a five or six months' fetus. I rested the body of the womb, with the fetus in it, on the palm of my right hand and arm, supporting and guiding the fundus with the four fingers. I cautiously inserted my hand in the pelvis, pushing the womb with the left hand. I replaced it in its natural position. I was very proud of this achievement, only to be disappointed in a few minutes. A severe pain came on, and the womb and baby (in it) shot out like a pebble from a sling. I replaced them three or four times, and even tried to hold them *in situ* when the pain came on; but it was no use—out it would shoot at every pain. The time had come for me to call for Aunt Nancy's assistance. I made her grasp the fundus and body of the womb with both hands—it still lay out on the bed. I then proceeded to dilate the os until I could get a firm grasp on the head, and, using my fingers as forceps, I at last succeeded in delivering the dead fetus, and then the after-birth. All this was done without any anesthetic, but whisky as a stimulant. Anesthetics were not used in those days but for surgical cases.

After the removal of its contents I replaced the womb, and for fear an after-pain might shoot it out again I took Lizzie's finest petticoat, tore it into rags, and packed the vagina as tightly as it was possible to get it. I think I used up nearly the whole of Lizzie's petticoat, for which I don't think she ever forgave me.

In spite of uterine and vaginal douches of Darby's Prophylactic and hot water, severe peritonitis supervened, and in four days her abdomen was the shape of a potato-hill. I put a blister all over the abdomen, kept her bowels open with calomel and salines, kept up the antiseptic douches, and procured rest and sleep with opium.

Lizzie made a good recovery. I left the State (Virginia) soon afterwards, entering the Confederate army, but I have learned that the woman has had several children since, and none of these troubles.

A moral for young physicians: Rely upon your own resources, think for yourself, and, believing you are right, go ahead. This advice I give, however, only to well-educated, thorough physicians—thorough in your anatomy, physiology, and, as in this case, obstetrics. In an hour I could have gotten my father and Dr. Wm. Smith, both distinguished physicians in their day, but I felt that if they came to my assistance they could not do anything more than what I thought was the proper thing to do. They could have done just what I did, and they *would have gotten all the credit;* whereas, when I told them what I did do, and assured them the woman was not dead, they said, "Well, she will die; BUT, if she gets well we shall think, with more experience and *strict attention to business*, you may in time make *a right sharp doctor*."

